# Sympathovagal Balance and Body Shape Index in Elite and Amateur Athletes: The Relationship Unraveled

**DOI:** 10.7759/cureus.45408

**Published:** 2023-09-17

**Authors:** Ruchi Kothari, Gaurav Mittal, Prashanth A, Yogesh S, Prince K Verma, Amisha Palande

**Affiliations:** 1 Physiology, Mahatma Gandhi Institute of Medical Sciences, Wardha, IND; 2 Research, Students Network Organization, Mumbai, IND; 3 Research and Development, Rotaract Club of Indian Medicos, Mumbai, IND; 4 Internal Medicine, Madras Medical College and Rajiv Gandhi Government General Hospital, Chennai, IND; 5 Physiology, Baba Raghav Das Medical College, Gorakhpur, IND; 6 Physiology, Terna Medical College, Mumbai, IND

**Keywords:** body shape index, heart rate variability, ergometer, treadmill, elite athletes, amateur athlete

## Abstract

Background

Heart rate variability (HRV) is one piece among a complex network of adaptations existent in athletes that help them gain a better understanding of their own physiology. Sympathovagal balance is one of the spectral components of HRV analysis and is used to assess the frequently changing oscillations of a healthy heart, which can help in gauging the response of cardiac function towards physiological stress during exercise. This index is extensively used in appraising cardiac autonomic modulation. An evaluation of body composition in athletes has become a critical consideration when tracking HRV, as it helps practitioners understand the role of the autonomic nervous system (ANS) in obesity. The body shape index (BSI), which is based on waist circumference (WC), is an anthropometric parameter with decent predictive ability when measuring centripetal obesity. In this regard, the current study is an attempt to unravel the relationship between BSI and sympathovagal balance during exercise performed on two different instruments (treadmill and ergometer) by elite and amateur athletes.

Methods

It was an observational case-control study that included 30 elite and 120 amateur athletes. Symptom-limited exercise testing was performed by athletes on a motorized treadmill and ergometer in the sports physiology laboratory of a rural medical college in central India. Different anthropometric parameters like BSI and body surface area (BSA) were also recorded. Short-term HRV extracted from electrocardiogram (ECG) recordings was obtained using the Power Lab system and HRV analysis by LabChart software.

Results

The sympathovagal ratio, i.e., ratio of low frequency (LF) to high frequency (HF) in elite and amateur male populations showed a higher value than that in females, indicating a dominant sympathetic response in the males. There was a significant (p=0.042) positive correlation (r=0.24) between BSI and LF/HF Ratio in amateur females during treadmill exercise, whereas a significant (p=0.049) negative correlation (r=-0.27) was obtained in amateur males during ergometer exercise. Hence, increased weight and BSI were found to be associated with high sympathetic dominance, indicating a sympathovagal imbalance.

Conclusion

We attempted to explore the interaction between BSI and LF/HF during exercise performed on two different instruments (treadmill and ergometer) by elite and amateur athletes, which can help in testing the response of cardiac function to stress experienced during exercise. The study's uniqueness stems from discovering the relationship between BSI and HRV and how this relationship impacts sports performance. BSI measurement in athletes, both elite and amateur, allows for the assessment and forecasting of potential autonomic activity under exercise-induced stress by linking HRV with BSI.

## Introduction

Not all humans are genetically adapted for the sustained, extreme aerobic efforts that athletes endure. Specifically, elite athletes tend to have superior cardiovascular physiology when compared to the average person [1]. Athletes nowadays have access to formerly unthinkable metrics and statistics to help them make decisions about their preparation for a competition, performance, and recovery strategies. In the contemporary, fast-evolving arena of athlete analytics, heart rate variability (HRV) continues to be a primarily employed tool, particularly for elite athletes [2]. The physiological basis of changing oscillations in a healthy heart, which is manifested as HRV, is considered to be an outcome of an individual’s autonomic nervous system (ANS). Frequency domain (or spectral analysis) measures like sympathovagal balance denote the contribution of various frequencies to the overall variability of the signal [3].

Driven by ease, the accurate interpretation and clinical applications of HRV metrics have drastically increased, making the use of HRV more common and widespread. It has now become compulsory and necessary for athletes to track their HRV data by taking measurements first thing in the morning [4]. While the avenues for upgrading athletic performance are abundant, so are the potential pitfalls. This has led to an increase in the availability and use of HRV metrics in the wide-ranging athletic population and may be particularly useful to elites in comparison to amateurs. Elite athletes generally exhibit a greater resting HRV than healthy individuals [5,6].

The use of HRV in monitoring the physiology of athletes is in its early days. It has become critical to evaluate body composition in athletes when tracking HRV in order to better understand the role of the ANS in obesity. Thus, an understanding of the different variables and the complex physiological interactions that produce differing patterns of HRV in athletes can help improve the interpretation of the data as well as give insights into improving their performance in terms of cardiovascular efficiency. Body shape index (BSI), which is derived from waist circumference (WC, independently from BMI), is an anthropometric parameter that is considered to be a better index of an individual’s body composition and has a decent predictive ability with respect to abdominal obesity.

Although some research has been performed in the past to determine the HRV indices pre- and post-exercise or in the resting state, there is a lack of literature on the same parameters in athletes during exercise [7,8]. Previous studies have investigated HRV indices in athletes but not with reference to amateur and elite athletes [9,10].

Due to scant data on body composition indices such as BSI in athletes and conflicting results in past research on HRV among athletes, this study was planned to unravel the relationship between BSI and sympathovagal balance during exercise performed on two different instruments (treadmill and ergometer) by elite and amateur athletes and to investigate the extent to which ANS contributes to obesity.

## Materials and methods

Study design and setting

This was an observational, cross-sectional study. The Strengthening the Reporting of Observational Studies in Epidemiology (STROBE) guidelines for the case-control study were used for reporting and preparing the manuscript. The study was carried out in the sports physiology laboratory in the department of physiology of a medical college in central India. 

Study participants

A total of 150 participants were recruited for the study. The sample size was estimated using OpenEpi 3.01 statistical software (Centers for Disease Control and Prevention (CDC), Atlanta) with the assumptions as the confidence level of 95%, alpha of 0.05, and power of study as 80%. To give the study more credibility and authenticity the case-to-control ratio was kept at 1:4. Hence, there were 30 cases and 120 controls. In this study, only pure athletes were recruited and were further classified into elite and amateur ones [11]. The elite athletes were considered as cases and amateur ones as controls in order to compare the anthropometric and cardiovascular parameters between them. They were grouped on the basis of the selection criteria. 

Each participant in this study provided informed consent, or they chose not to. MGIMS/IEC/PHY/127/2017 was approved by the Mahatma Gandhi Institute of Medical Sciences' Institutional Ethics Committee for Research on Human Subjects.

Selection criteria

As per the definition of an athlete by the American Heart Association, “a participant in an organized team or individual sports that place a high importance on excellence and performance, regular competition against other athletes, and involve some type of systematic training (typically intensive)”, subjects were chosen after proper inquiry and history taking [11].

Further, the subjects who were deemed as athletes as per the selection criteria were classified into elite ones and others were considered as amateur ones. Elite athletes were those who competed at the collegiate level, and at the national level, won medals, were seasoned professionals who held records, and trained frequently [12].

Athletes between the age of 18-40 years and those who gave informed written consent were included in the study. Asthma, emphysema, chronic obstructive pulmonary disease, chronic hypertension, diabetes, musculoskeletal diseases, cardiovascular conditions, diagnosed psychiatric disorders, subjects experiencing musculoskeletal pain on the day of the test, and those who refused to consent were among the diseases that were excluded from the study (exclusion criteria). Only after assessment and clearance by the institutional ethics committee did the study get underway. 

Data sources and measurement of variables

Anthropometric Variables

The subjects' age was recorded in years. The individuals' standing height (in cm) was measured while they were barefoot and stood with their heels together. Weight (in kg) was measured when the subject was standing, wearing just light clothing, and barefoot. Over bare skin or light knickers, WC was measured using a measuring tape.

The individual was in a standing position when measurement sites were taken. In the horizontal plane, the WC was measured midway between the iliac crest and the lower border of the ribs. Two measurements were taken and recorded to the closest 0.5 cm. A third measurement was done if there was more than 2 cm of difference between the first two. It was determined what was closest in terms of the two metrics. The Phoenix Height Weight Body Mass Index machine (Model: PBMI-200) measured BMI [13].

WC was used to create the BSI, which is considered to be a superior index. BSI was calculated using the formula below [13].

$BSI = WC/ [(BMI)^{2/3} * (Height)^{1/2}]$ 

Body surface area (BSA) was calculated using the formula below [13].

$BSA = 0.20247 * Height^{0.725} * Weight^{0.425}$

Heart Rate Variability

HRV was recorded using the Power Lab system and analyzed by the LabChart software by AD Instruments, Australia. This system gave live wireless electrocardiogram (ECG) along with a recording of HRV parameters while the subject was maximally exercising on the treadmill and cycle ergometer. Fast Fourier transformation is used to extract frequency domain (or spectrum analysis) estimates of HRV from time domain data, which show the relative contributions of various frequencies to the signal's overall variability [14]. Very low frequency (VLF, 0.04 Hz), low frequency (LF, 0.04-0.15 Hz), and high frequency (HF, 0.15-0.4 Hz) spikes are the most often seen. According to conventional wisdom, HF power represents parasympathetic activity while LF power represents sympathetic activity [9]. The ratio of LF to HF is thought to be a reflection of sympathovagal balance [15]. HRV recordings were separately done when the subject was exercising on the motorized treadmill and cycle ergometer (Aerofit AF 101, Nityasach Fitness Pvt Ltd, Mumbai, India).

Statistical analysis

The collected data were input into an Excel spreadsheet, where the mean and standard deviation were calculated. The SPSS (Statistical Package for Social Sciences, Version 20.0, SPSS Inc, Chicago, Illinois, USA) was used to analyze the data. Using Pearson correlation, the study's variables were correlated. The information was normally distributed. The t-test was used to determine the significance of the correlation coefficient value "r" between two quantitative variables, which might be positive (direct correlation) or negative (inverse correlation). The significance threshold remained at p< 0.05.

## Results

Out of 120 amateur athletes, 50 (41.7%) were males and 70 (58.3%) were females. Of a total of 30 elite athletes, 17 (56%) were males and 13 (44%) were females. Male elite athletes had a mean age of 20.94 ± 2.75, while female elite athletes had a mean age of 22.17 ± 3.30. Amateur males had a mean age of 21.06 ± 3.02 while amateur females had a mean age of 22.50 ± 6.25. Tables 1, 2 list various demographic information along with anthropometric and cardiovascular readings of the study participants. As per the tables, it can be deduced that there was a statistically significant difference among males in the mean values of BMI (p=0.021) and BSI (p=0.012). However, there was no statistically significant difference in the remaining parameters in both the groups and the genders.

**Table 1:** Descriptive statistics of the male subjects BSA: Body surface area; BSI: Body shape index; LF: Low frequency; HF: High frequency; WC: Waist circumference; HR: Heart rate

Parameter	Elite Males (n = 17)				Amateur Males (n = 50)			
	Mean	Standard Deviation	95% confidence interval for mean		Mean	Standard Deviation	95% confidence interval for mean	
			Lower bound	Upper bound			Lower bound	Upper bound
Height (m)	1.75	0.04	1.73	1.76	1.7	0.07	1.68	1.72
Resting HR (beats/min)	80.18	8.09	76.33	84.02	84.06	10.87	81.05	87.07
Weight (kg)	65.65	10.68	60.57	70.72	68.02	11.04	64.96	71.08
WC (cm)	84	5.83	81.23	86.77	82.24	18.42	77.14	87.34
BMI (kg/m²)	21.5	2.83	20.16	22.84	23.66	4.32	22.46	24.85
BSA (m²)	1.79	0.14	1.73	1.86	1.78	0.13	1.74	1.82
BSI	0.083	0.003	0.08	0.085	0.077	0.016	0.073	0.082
LF/HF Ratio (Treadmill)	1.3	1.05	0.8	1.8	0.87	1.26	0.52	1.22
LF/HF Ratio (Ergometer)	2.77	2.25	1.23	0.63	2.79	4.91	1.43	4.16

**Table 2:** Descriptive statistics of the female subjects BSA: Body surface area; BSI: Body shape index; LF: Low frequency; HF: High frequency; WC: Waist circumference; HR: Heart rate

Parameter	Elite Females (n = 13)				Amateur Females (n = 70)			
	Mean	Standard Deviation	95% confidence interval for mean		Mean	Standard Deviation	95% confidence interval for mean	
			Lower bound	Upper bound			Lower bound	Upper bound
Height (m)	1.62	0.07	1.58	1.65	1.61	0.04	1.6	1.63
Resting HR (beats/min)	84.17	13.86	76.32	92.01	89.5	10.75	81.05	87.07
Weight (kg)	57	8.63	52.11	61.89	58.91	8.43	55.99	61.83
WC (cm)	85.08	6.88	81.19	88.98	87.41	8.41	84.49	90.32
BMI (kg/m²)	21.82	3	20.13	23.52	22.59	3.13	21.51	23.68
BSA (m²)	1.6	0.13	1.52	1.67	1.62	0.11	1.58	1.65
BSI	0.086	0.004	0.084	0.088	0.087	0.007	0.084	0.089
LF/HF Ratio (Treadmill)	0.55	0.26	0.4	0.69	0.71	1.1	0.33	1.09
LF/HF Ratio (Ergometer)	0.95	0.84	0.48	1.43	1.31	1.14	0.91	1.7

Further correlation analysis was also performed, primarily between sympathovagal balance and BSI, in elite and amateur male and female athletes. As the recordings were taken using a treadmill and ergometer, two separate datasets were obtained, as depicted in Tables 3, 4. A statistically weak significant positive relationship (r=0.24, p=0.042) was found between the two parameters in amateur females on treadmill exercise and a weak negative correlation (p=-0.27, p=0.049) which was statistically significant was obtained among amateur males upon ergometer exercise. There was a strong negative correlation between LF/HF ratio and BSI in elite female athletes but it did not reach statistical significance. 

**Table 3:** Correlation of parameters obtained on treadmill BSI: Body shape index; S: Significant; NS: Not significant; LF: Low frequency; HF: High frequency

Study group	BSI (Mean ± Standard Deviation)	LF/HF	r value	P value
Amateur males	0.077 ± 0.016	0.87 ± 1.26	-0.10	0.48(NS)
Elite males	0.083 ± 0.003	1.30 ± 1.05	0.107	0.662 (NS)
Amateur females	0.087 ± 0.007	0.71 ± 1.10	0.24	0.042 (S)
Elite females	0.086 ± 0.004	0.55 ± 0.26	- 0.11	0.696 (NS)

**Table 4:** Correlation of parameters obtained on ergometer BSI: Body shape index; S: Significant; NS: Not significant; LF: Low frequency; HF: High frequency

Study group	BSI (Mean ± Standard Deviation)	LF/HF	r value	P value
Amateur males	0.077 ± 0.016	2.79 ± 4.91	- 0.27	0.049 (S)
Elite males	0.083 ± 0.003	2.77 ± 2.25	0.13	0.595 (NS)
Amateur females	0.087 ± 0.007	1.31 ± 1.14	-0.11	0.357 (NS)
Elite females	0.086 ± 0.004	0.95 ± 0.94	-0.42	0.119 (NS)

## Discussion

HRV indices, particularly sympathovagal balance, are viewed as measures of intricate cardiac autonomic modulations of an athletic heart. Alterations of HR are known to contribute to improvements in predicting performance, enhancing training and competition strategies, and refining the science of sports physiology. Earlier research implicating HRV to sports has focused majorly on endurance sports. Lundstrom et al. found that greater HRV scores indicated better cardiovascular adaptations due to higher training intensities, favoring HRV as a measure to optimize individualized training in elite runners [1].

In spite of this, published data on professional athletes are uncommon; the majority of HRV research conducted so far has involved trained or recreational participants [16-18]. Elite athletes may react differentially to training pressures and subsequent recovery due to genetics and training experience [19,20]. Therefore, top athletes may use HRV in a number of ways as the data has the potential to improve their competitive performance to the fullest.

There is a widespread misperception among sports physiologists that there is a direct linear link between vagal-related indices of HRV and the parasympathetic impact on heart rate (HR) when using HRV to determine autonomic health. Actually, a quadratic connection exists [21]. This implies that vagal-related HRV indices are decreased at both low (high HR) and high (low HR) levels of vagal tone. For instance, elite athletes who have received proper training typically have both a low resting HR and elevated HRV indices [22]. However, many athletes with low resting heart rates have also been found to have lower HRV [23]. The primary mechanism is probably myocyte-level acetylcholine receptor saturation: elevated vagal tone may result in persistent parasympathetic regulation of the sinus node, which may lower HRV [24].

In the current study, during exercise, the HF (vagal) component increased significantly, in elite athletes whereas the LF component and the LF/HF ratio decreased as compared to amateur counterparts which reflects parasympathetic dominance in the elite athletes. This finding goes in agreement to the well documented fact that exercise leads to an increase in heart rate to meet increased metabolic demands, while frequency HRV measures decrease dramatically [10,25,26].

Owing to the wealth of information and metrics available to athletes, there is growing interest in physiological forecasting, or predicting results or projecting outcomes based on a number of variables that may be obtained via wireless HRV technology during exercise. This unique modality of HRV measurement was a major strength of this study. According to Treff et al., this technique may also prove to help in enhancing the overall performance of athletes by quantifying stress and recovery [27]. Highly trained elite athletes returned to baseline more quickly than amateur athletes in the current study as well.

As per the existing literature exploring HRV as a way to assess cardiometabolic risk, the sensitivity of HRV to body composition indices has been well-established [28]. So it is reasonable enough to assume that elite and amateur athletes may likewise experience an impact on their HRV due to an index like BSI. It has also been noted in amateur athletes that there were improvements in body composition and strength when training was guided by HRV. With regard to BSI values, the elite and amateur females did not differ significantly from each other while the amateur males had lower BSI as compared to elite males. However, the female athletes had a higher BSI than their counterparts [28].

The sympatho-vagal ratio in the elite and amateur male population showed a higher value than that in females indicating a dominant sympathetic response. There was a weak significant positive correlation between BSI and LF/HF Ratio in amateur females on treadmill exercise whereas a weakly significant negative correlation was obtained in amateur males on ergometer exercise. 

Like every research project, this one had some limitations as well. The sample size was limited due to the brief duration of this short-term research. The age range was narrowed in order to accomplish the achievable target group of athletes. The data was collected at a specific point in time as it wasn’t a longitudinal study. This design does not allow for causal relationships to be established or changes in readings over time to be examined. HRV testing has several disadvantages as well, such as the time commitment and participation of the individual throughout the test. Recruiting interested volunteers for this kind of study was extremely challenging. Hence, prescribing them or guiding them to get their autonomic and anthropometric parameters tested at a regular basis would pave way for further progress and outcomes in the field. 

## Conclusions

To conclude, the use of the HRV index like sympathovagal balance represents an inexpensive alternative to evaluate and predict possible autonomic activity during exercise. It may help improve the performance of both the elite and the amateur athletes. This article further highlights that HRV monitoring along with BSI measurement in elites is required to understand their unique individual HRV fingerprint. This study demonstrated how increases and decreases in HRV relate to variations in BSI in elite and amateur athletes. 
